# The Impact of a School Dog on Children’s Social Inclusion and Social Climate in a School Class

**DOI:** 10.3390/ejihpe14010001

**Published:** 2023-12-19

**Authors:** Mona M. Mombeck, Timm Albers

**Affiliations:** Inclusive Pedagogy, Institute for Educational Science, University of Paderborn, 33098 Paderborn, Germany; timm.albers@upb.de

**Keywords:** animal-assisted, social inclusion, social climate, child development, animal-assisted pedagogy, one health, wellbeing

## Abstract

Animal-assisted pedagogy is well known in classroom practice, but scientific evidence of its impact on teaching and learning conditions is still lacking. At the same time, the biggest challenge in education systems worldwide is the social inclusion of students. In a pre–post design, 30 heterogeneous students (16 f/14 m) from four different school classes (grades 5–8) of two secondary schools and one grammar school were interviewed (in a problem-centered interview) about their social inclusion and their social climate in class before and after being taught selected subjects with a school dog for one school term. At the second measurement point, participants were also asked about their perception of animal-assisted pedagogy. The qualitative data analysis (Kuckartz) showed that the presence of a dog leads to an improved social climate, more social integration and to a change in social roles; therefore, we discussed our findings in the context of role theory (Krappmann). In addition, we found that the mutual perception of the other students and the teacher changes to a more positive and friendlier image. Through animal-assisted pedagogy, a new social role is added to the classroom, where caring and bonding are prioritized. Social interaction and norms are influenced and stereotypical and individual roles can be changed. Therefore, animal-assisted pedagogy can be key to promoting social inclusion in the school environment.

## 1. Introduction

Enhancing the social inclusion of all children presents a distinct challenge within the inclusive school environment. In particular, children with special educational needs are at a high risk of exclusion [1]. This is reflected in the fact that children with special educational needs experience less acceptance and more rejection from their fellow students [2,3,4,5]. As a result, students with special educational needs feel less socially integrated [2], have a less favorable perception of the class environment, and feel less accepted by the teaching staff [6]. According to Schürer [5], the highest risk of social exclusion is among children with special educational needs focused on emotional and social development, who rate their own social inclusion, class climate, and school self-concept significantly more negatively than their classmates [7]. Special educational needs, however, do not represent the sole predictors of an increased risk of exclusion. Weak academic performance, as well as behavioral challenges, likewise have a negative correlation with social participation (even without special educational needs being identified) [8]. Another predictor for social exclusion could be the “labeling effect” created by the term “special educational needs” [9]. Crede et al. [7] argue that the exclusion of children is because students choose their social interaction partners based on characteristics that they share. It can therefore be assumed that social integration is particularly difficult to achieve in heterogeneous classes. While studies reveal that social participation can be strengthened through a good classroom climate and inclusive pedagogical practices [3,10], there is still a lack of concepts and opportunities for promoting inclusive social structures in school classrooms. Simultaneously, social participation is based on the foundation of mutual friendships, relationships, and acceptance. It is an independent and voluntary choice made by individuals, which makes it challenging to influence through predefined concepts. Consequently, the significance of strategies aimed at fostering social inclusion becomes even more pronounced [11].

With this in mind, more and more teachers have been including animals, especially dogs, to promote social participation over the last decades. During these interventions, the dog is mainly present and takes on a more passive role [12]. 

The concept of animal-assisted pedagogy includes establishing rules for dealing with the dog, responding to the dog’s needs, and maintaining the dog’s boundaries [12]. Positive implications can be confirmed in initial studies for the school setting. For example, Kotrschal and Ortbauer [13] observed a decrease in aggressive behavior, more attention towards the teacher, and fewer behavioral extremes in a heterogeneous elementary school first grade if daily lessons were taught with a dog for three months [13]. Hergovich et al. arrived at similar conclusions in a compareable setting [14]. The prerequisites for social participation of students can also improve, since animal-assisted pedagogy significantly reduces the stress level of students, especially for children with special educational needs [15]. However, the scientific validation of its influence on teaching and learning conditions is still insufficient. Neither children’s perceptions of everyday animal-assisted pedagogy in class were investigated, nor are there any data on school dogs involved in secondary schools.

A larger number of studies deal with animal-assisted out-of-school interventions and animal-assisted prevention programs. However, in such programs, there are often fewer children taking part than are present in a regular school class, meaning that the setting does not correspond to the reality of school, while the intervention, e.g., the interaction with the animal and the intensity and duration of the contact with the animal, also differ significantly. Yet, the following results should be viewed as suggestive of the potential implications of animal-assisted pedagogy for social participation in the heterogeneous school setting. 

Brelsford et al. [16] conclude in their meta-analysis that animal-assisted interventions have a positive impact on socio-emotional behavior. However, the authors criticize that the research results are unclear due to the heterogeneity of the studies, which makes interpretation difficult and reduces the significance. Previously, Clarke [17] revealed that both in-school and out-of-school programs and interventions have predominantly positive effects on the emotional and social development of children and adolescents ages 4–20. Recent studies of programs also confirm that interaction with animals helps children to recognize their own emotions as well as the emotions of others and show that animal-assisted interventions contribute to the development of socio-emotional skills [18].

Work on specific target groups has shown further positive effects of animal-assisted interventions. The promotion of socio-emotional skills through animal-assisted interventions in children with autism spectrum disorder is a good example, resulting in significant improvements in social functioning, increases in social interaction, as well as the development of emotional and social skills [19,20,21].

Moreover, research reveals the positive effects of animals—in non-school settings such as therapy or regarding pets—on the predictors and conditions of socio-emotional competence. Being around and in contact with animals is associated with socio-emotional health, reinforcement of social networks, rest, and improved socio-emotional behaviors [12,22,23,24,25]. These findings match with current findings that the impacts of animal-assisted interventions can be attributed to the activation of the oxytocin system, which is decisively responsible for the implementation of social action [23,26]. It is remarkable that children exhibiting insecure-avoidant attachment patterns, in particular, benefit from the presence of a dog in stress tests, which results in lower cortisol levels [27].

Teachers have a key function in reducing exclusion and promoting inclusion. They should be sensitive to social processes and group dynamics and use proactive strategies to prevent social exclusion before it occurs [28].

Given the findings of previous studies, animal-assisted pedagogy seems to be promising when it comes to promoting social inclusion. However, it remains to be evaluated to what extent these results can be transferred to the heterogeneous school setting, how pupils perceive animal-assisted pedagogy, and how teaching with a dog affects social inclusion of pupils. We attempt to answer these questions in this study. The results are discussed concerning role theory to contextualize the impact of animal-assisted pedagogy on school structures and interaction patterns.

## 2. Materials and Methods

### 2.1. Objective and Analytical Framework

The objective of this research is to evaluate the implications of animal-assisted education on the social participation of students and the social climate of school classes. With this in mind, we aim to answer the following questions [29].

Question 1: How does animal-assisted pedagogy impact social participation opportunities and social climate? 

Question 2: What potential does animal-assisted pedagogy have for the participation opportunities and social climate of heterogeneous groups?

With our research, we focus on the subjective theories and cognitions of students, allowing us to map subjective contexts of meaning and constructions of reality [30,31]. Subjective theories are regarded as the basis for orientation and action for individuals [32]. They serve definitional, explanatory, predictive, and planning functions for the individual, and thus form the basis for his or her actions [33]. The theoretical foundation for the concept of subjective theories draws from symbolic interactionism [29,34,35]. As the initial analysis of the results provides indications that pupils show a change in role behavior, role theory by Krappmann [36] was used to discuss the findings in an in-depth analysis.

The role theory focuses on interpersonal negotiation processes which depend on social roles, role action, and norms [36]. In this respect, the formation of an individual’s identity is both the result and the condition of the negotiation process. By taking a social role (*role-taking*), an individual has the opportunity to shape this role to suit his or her identity (*rolemaking*), whereas, on the other hand, each social role shapes the person’s own identity, too. *Role actions*—that is, social negotiation processes or social interaction—are conditioned not only by the individual’s role design but also by the interaction partner, their expectations and needs, and by the norms in a setting. Social roles and social interaction are determined by social norms.

In the school context, this means that a child acts in the social role of a *student* (*roletaking and rolemaking*) (and several other social roles, such as *best friend*, a child with *special educational needs*…) and interacts with other persons in their social roles of being a *student* and *teacher.* This interaction in specific roles is called *role acting*, which is driven by norms typical for school, such as performance and selection [37].

### 2.2. Method and Design

**Figure 1:**
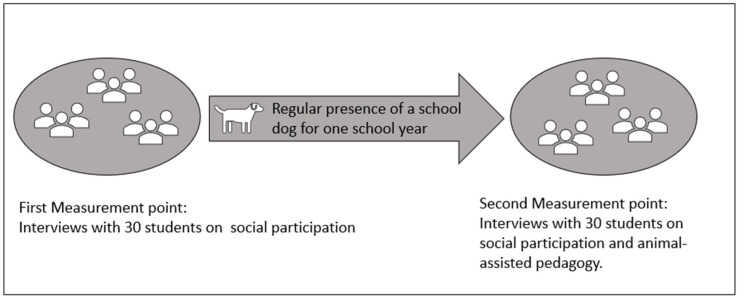
Design of survey.

Thirty students were interviewed in a pre–post design (Figure 1) using problem-centered interviews, a guideline-based, theory-generating interview method in which the interviewer both verifies deductive prior knowledge and generates inductive knowledge through the interpretation of the material to gain knowledge [38]. The participants were questioned about their social participation and the social climate in their class (Topic Block A). At the second measurement point, an interview was also conducted to determine the specifics of animal-assisted teaching (Topic Block B). The intervention period between the measurement points was one school semester (approx. 6 months). During this time, the children were taught in the presence of a dog one to three times a week for 90 min. The design and the implementation met the conditions for research on animal-assisted interventions [39]: The study was conducted in a natural school environment and the safety and well-being of both humans and animals were ensured. Two of the four dogs had been involved as school dogs before. The other two dogs were gradually habituated to the school building, classroom, and teaching situation. All human–dog teams were appropriately trained for animal-assisted pedagogy. The selection of the human–dog teams was carried out through conversations with the teachers to determine whether their attitude and the implementation of animal-assisted pedagogy align with the guidelines of the Quality Network for School Support Dogs [40], at the time of data collection [41]. In addition, it was examined whether the animal-assisted teaching methods complied with the guidelines of the Ministry for School and Further Education of the State of North Rhine-Westphalia on animal-assisted pedagogy with dogs [42]. The study design was approved by the Ethics Council of the University of Paderborn (Germany).

### 2.3. Sampling

Thirty children were selected according to Helferrich’s sampling method, a three-stage process used to define a sample [43]. First, a narrow scope of the group of interest was defined: four school classes with a heterogeneous student body in which animal-assisted teaching had not yet taken place. They were an eighth, a fifth, and a sixth-grade class from two different comprehensive schools, as well as a fifth-grade class from a grammar school (all schools are located in North Rhine-Westphalia, Germany). Further selection criteria were that teachers and their dogs had a proven qualification for animal-assisted work (specialist training or training as a human–dog team with verification that the dog is healthy and suitable). In the second step, a broad variety of 30 pupils were selected within the different classes. Approximately equal numbers of children were chosen from each class. The 2017/2018 average distribution of the diversity dimensions—nationality, special educational needs, gender, and migration background—in schools in North Rhine-Westphalia [44] was used as a reference for this selection. This selection does not conform to a broad inclusion approach but was intended to expand the scope. These diversity dimensions are meant to ensure a wide variation but play a minor role in the analysis, resulting in a sample of 30 children that can be defined as follows (third step): 16 female, 14 male, 11 pupils with international background (with an immigration history, with German citizenship), 4 foreign children and young people who do not have German citizenship, and 2 children with diagnosed special educational needs in the area of emotional and social development. The average age (mean) at the first measurement point was 12.52 years (SD 1.74 years). The dogs involved were always dogs kept by the teachers and varied in breed, age, and gender: a Labrador (female, 4 years old), a Border Collie (male, 3 years old), a West Highland Terrier (male, 5 years old) and an Old English Sheepdog (female, 3 years old). 

### 2.4. Analysis

Simple transcription rules, following the content semantic transcription guidelines, were selected for the preparation of the data [45], and the transcription software F4 was used. The researcher also anonymized the data at this point. The qualitative content analysis, according to Kuckartz [46] was applied for the analysis and conducted with the use of QDA software (MAXQDA Analytics Pro 2018). 

**Figure 2:**
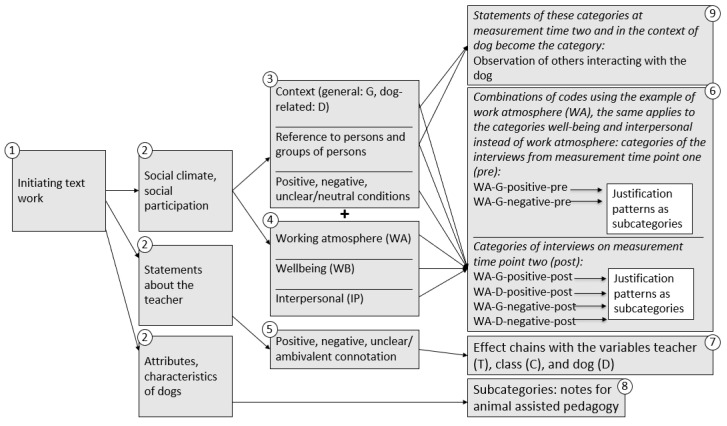
Origin of the categories.

The forms of analysis used included the content structuring form and an analysis form following the evaluative form [46]. The procedure is detailed in the work of Mombeck 2022 [29], which is why only the short form of the work steps is presented here. The analysis resulted in the following work packages, also illustrated in Figure 2 [29].

1. Initiating text work with the aid of the postscripts and the full transcripts of the problem-centered interviews.
2. Coding of pre-formulated main categories: (A) Social climate and social participation, (B) Learners’ perceptions of the teacher, and (C) Comments on Attributes and characteristics of dogs, regardless of the topic of social climate.
3. Category formation: Coding along the main categories A in (a) Evaluation: Is the statement positive, i.e., advocating or pointing out advantages or positive developments regarding aspects of one of the central themes? (animal-assisted pedagogy, social interaction), neutral or negative, criticizing, highlighting a problem? (positive, problematic, and unclear/neutral) and in (b) Context: Is a direct reference to animal-assisted pedagogy made or not? (dog, no dog). Moreover, the main category A was categorized in a differentiated manner, in (c) References (to oneself, to individuals (others), and everyone; that is to say, the whole class and generally formulated statements).
4. Formation of subcategories along the main category A: Coding as Working atmosphere, Interpersonal, and Well-being.
5. Category formation: Coding along the main category B in Assessment (positive, problematic, and unclear/neutral).
6. Combination of subcategories and emphasis onJustification patterns as further subcategories along the categories Working atmosphere, Interpersonal, and Well-being and further differentiation of the justification patterns if necessary. Two predominant and frequently encountered categories for justification/explanation are “actions for the dog” and “actions through the dog”.
7. The statements about the teacher from the post-interviews are coded into the Impact chains (Effect chains) category. The impact refers to the interaction of teacher–dog—class (T-D-C combined in various ways).
8. Along the main category C: The Statements about attributes and the Meaning attributions to dogs are coded into three thematic subcategories.
9. Expanding on the subcategory Individuals and Everyone as well as Context dog at the second measurement point, selected statements are coded as the category Observations of others interacting with the dog.

To answer the question regarding the effects of a school dog on the social climate and social participation (results in part 1, chapter 3.1.1.–3.1.4), the results of the qualitative content analysis are presented in the main categories of *Working atmosphere*, *Well-being*, *Interpersonal* and the category *Perception of the teacher*. The subcategories *Working atmosphere*, *Well-being*, and *Interpersonal* include the *positive* and *negative connotations* of the statements of these categories, as well as justification patterns in the statements (*justification*) and the persons and groups of persons to which each statement is related (*self*, *others*, *class community*). The statements on the different categories are examined to identify any change between pre-and post-interview.

To determine the potential of animal-assisted pedagogy (results in part 2, chapter 3.2), the categories and subcategories of *attributes and characteristics of dogs*, the category *observations of others interacting with the dog,* and the categories *effect chains* with the variables *class*, *teacher* and *dog* are analyzed.

## 3. Results

### 3.1. Part 1: Implications of Animal-Assisted Pedagogy for Social Participation and Social Climate

#### 3.1.1. Working Atmosphere

Statements of the category *Positive working atmosphere* are rarely cited at the first measurement point (5), but are cited significantly more frequently after the intervention (3 in the *general context*/27 in the *dog context*). It appears that the participant’s perception of the working atmosphere has fundamentally changed, likely due to the animal-assisted pedagogy. According to the participants, the presence of the dog leads to a better learning environment, increased calmness, discipline/respect, and cleanliness in the class, which makes it easier to concentrate. The statements can be divided into the categories of *Actions for the dog* and *Actions through the dog’s presence* (according to step 6 of the category development).

*Actions for the dog* refer to students consciously behaving in a way that meets the needs of the animal and makes it feel comfortable. The improved working atmosphere becomes a by-product of this.

“So yes: we’ve been very considerate of [dog’s name] there as much as possible. And yes, things got much calmer because everyone simply listens to the rules and cares for the dog”

(F8_28, Group 2, Segment 52).

Behavior that was caused by the dog’s presence (*actions through the dog*) describes statements where the dog being present results in improved concentration or calm behavioral patterns that positively affect the working atmosphere.

“Well, I just think it’s great (…). I can sometimes concentrate better when there’s [dog’s name] next to me”

(H6_5, Group 2, Segment 28).

Statements bearing negative connotations in this category (*negative working atmosphere*) are mentioned at both measurement points. The reasons for this are behavioral problems of individual students, restlessness, and increased noise levels. The frequency of negative statements in the *general context* decreases (pre 17, post 4 in the *general context*), along with a reduction in the severity and the dramatic nature of the statements.

Pre: “Yes, because we don’t behave all the time”

(F5_4, Group 1, Segment 10).

Post: “So when the dog is around, there’s no stress really, but when there are other teachers around (who do not teach animal-assisted), there’s stress sometimes, and that’s still the same as always”

(F5_21, Group 2, Segment 3).

Negative statements regarding the *working atmosphere* with justification due to the animal-assisted setting are relatively high in number (12 in the *context of dog*) and are associated with having unfulfilled needs in terms of actions and behaviors related to the dog. Even minor or infrequent breaches of the rules set for the dog are considered a burden and an unfavorable working atmosphere because of the pity that children feel for the dog in such situations. Distractions due to the dog are also referred to as such.

“So, I can concentrate better because the class is quiet. However, I cannot concentrate so well when the dog is there because I always want to stroke the dog, and I also want him to develop trust in me”

(F5_12, Group 2, Segment 35).

#### 3.1.2. Well-Being and Uneasiness

Before the intervention, only a few positive statements were made about well-being (5), (“I feel good at the moment” S5_17, Group 1, Segment 6). At the second measurement point, only five participants comment positively on *well-being* in the *general context*. In contrast, almost all participants (29) cite well-being in connection *with the dog* at the second measurement point. The reasoning patterns (*justification*) in this category are particularly complex.

About the subcategories *self* and *others*, the well-being resulting from animal-assisted pedagogy is most often attributed to positive stimulation (26 participants): The dog provides fun, joy, and motivation.

“Because you feel somehow differently there, the room feels more alive because, in the class, you only move your hand, yeah. And then everything is just a little bit more alive”

(H6_21, Group 2, Segment 44).

“So, you look forward to doing math a lot more. I never really wanted to go to math before, it was boring, and I always wanted to just go home (laughs). But now I’m looking forward to math when (…) but only when the dog is there”

(F8_7, Group 2, Segment 33).

“So, the first time [dog’s name] was there, I was excited at first, I was happy the whole day, while the last few days I wasn’t excited because I already knew [dog’s name], I was just happy that she was with us. I was just happy all the time then, too. Whenever she was there”

(F5_13, Group 2, Segment 18).

In addition to these positive stimulations, the absence of negative stimulations through the dog’s presence also results in an increased sense of well-being (13 participants). This evokes feelings of happiness, contentment, and relaxation, which puts learners in a calmer and more serene state of mind. In brief, there is a reinforcement of positive stimuli as well as a reduction in negative stimuli:

“It’s just that when you’re a little bit stressed, for example (…) writing a paper or something (…) and then [dog’s name] is sort of lying there quite relaxed, then you also become sort of relaxed”

(S5_14, Group 2, Segment 63–65).

Experiences of self-efficacy in interacting with the dog also contribute to an increased sense of well-being on an individual level. For example, overcoming fear of the dog and interacting safely with dogs leads to a sense of well-being (in oneself and of others). In addition, the absence of external negative factors is cited, such as across-the-board disciplinary tasks by the teacher, which no longer occur since the introduction of animal-assisted teaching.

The well-being of the whole class (class community) is influenced by animal-assisted pedagogy, as it offers a new possibility of *identification*. The children in the class identify themselves as the “dog class” (four participants). The participants (24) describe a feeling of being at ease and well-being for several children or the whole class community.

“R (Respondent): I feel, like using a metaphor, for example, if you mix cocoa with milk now. Then it turns into a drink. And this drink certainly tastes good. And that’s the same feeling. I (Interviewer): Just a feeling of well-being. R: Yes. I: A little bit of enjoyment, relaxation too? R: A bit of relaxation (unintelligible). I: OK. Why is that so? Why do you get that feeling when there’s a dog around? R: I don’t exactly know, but it’s just that kind of feeling. It’s something I can only describe myself a little. I: OK. And you have this feeling? R: I think a few of us have, I’m not alone”

(F8_1, Group 2, Segment 48–56).

Feelings associated with *negative connotations* are mentioned by 4 participants at the first measurement point; *negative well-being* in the *general context* is mentioned by one participant at the second measurement point, although it is mentioned by 17 participants in the animal-assisted context. The feeling of uneasiness (*negative well-being*) stems from the concern that other children may feel uneasy in the animal-assisted setting. This concern originates from the fact that some children had voiced reservations or fear before the animal-assisted intervention began. Yet, they had agreed to the gradual introduction of animal-assisted pedagogy. These children were largely able to put aside any fears and are proud of their development (category *well-being*). Other children are unaware of this development or base their statement on the beginning of animal-assisted pedagogy.

“Yes, only some. Well, for example, Fe24 is a little frightened of dogs now, but generally, she’s also frightened of cats and stuff. That’s why, but she’s getting used to it, I think, anyway”

(F5_18, Group 2, Segment 72).

This explains the disparity between *positive well-being* about the *class* as a whole and *negative well-being* about *others*. This assumption can be confirmed by the fact that one’s uneasiness is rarely mentioned.

“Yes, so (…) I’m not as scared (…) anymore, doesn’t faze me as much”

(F8_7, Group 2, Segment 152–153).

If you ask those concerned whether they would therefore prefer to forego animal-assisted pedagogy, the answers given are no. Also contact that is too infrequent with the animal becomes a cause of uneasiness in rather rare cases.

“I was a bit sad because the dog never came to me”

(F8_30, Group 2, Segment 28–43).

#### 3.1.3. Interpersonal Dimension

Statements on interpersonal relationships (category *interpersonal*, e.g., mutual acceptance, friendships, class cohesion, interactions with others, and attempts at de-escalation) are named without direct reference to animal-assisted education (*general context*) both in the interviews before (27) and in the interviews after the intervention (25). The statements of 18 participants can be assigned to the subcategory which includes direct references to the animal-assisted setting (*context dog*). On the one hand, the dog is viewed as a unifying element, as pupils now have an interest that they share and a common goal of caring for the dog (10 participants).

“Because before, everybody kind of had their own goal. Like, for example, some wanted good grades, others wanted to be cool, and others just kept to themselves. And now, we all have our shared goal, that the dog stays here”

(F8_3, Group 2, Segment 62–64).

On the other hand, the dog’s presence is perceived as de-escalating. Because the participants want to show consideration for the dog, interpersonal conflicts occur less frequently and subside more quickly. Additionally, other children are perceived as less aggressive and more caring (9 participants):

“When the dog is around, there’s no stress really”

(F5_21, Group 2, Segment 3).

“There’s less conflict between people, between groups”

(F8_6, Group 2, Segment 2).

Negative expressions of the category *Interpersonal in a general context (Interpersonal-general context-negative-pre/post)* tend to be at high levels both before (23) and after the intervention (22). Bullying and the exclusion of individual children are frequently mentioned before the intervention (19). The reasons for this include dislike due to behavioral problems, physical appearance, or origin. The inability to regulate one’s own emotions also leads to exclusion and problematic interpersonal relationships. Not only active exclusion is mentioned but also the lack of positive actions and opportunities for participation (18). The exclusion of children is also attributed to unfavorable group dynamics (9), while another participant mentions that the class lacks cheerfulness, which leads to conflicts. 

Similar categories are observed at the second measurement point; the absence of positive actions (12), active negative actions (11), individual dislikes (9), and peer pressure (6). Although some of the results indicate that exclusions are less frequent, negative social aspects have not been eliminated.

Only two participants describe a negative influence of animal-assisted education on interpersonal dimensions. One participant describes jealousy due to the unequal animal contact and another participant considers defending the dog, even with violence, if it was mistreated by others.

#### 3.1.4. Perception of the Teacher

The teaching staff are perceived in different ways by the participants. The categories range from appreciating (*positive*: teachers are described as friendly, fair, close to the students, competent) (11), to the category *ambivalently/unchanged* (the description either lacks a clear assessment and cannot be interpreted as positive or negative, or nothing has changed) (6), to *negative* (10) (teachers are described as unfriendly, unfair or too strict). If teachers are already perceived positively before the intervention, participants also describe them as friendly after the intervention. Teaching personnel who were initially perceived in a negative light before commencing teaching alongside a canine companion exhibit a significantly higher frequency of positive attributes ascribed to them during the subsequent measurement point assessment, with the result that 26 participants rated the teaching staff positively after the intervention and only one person still makes a negative statement. Eight participants make statements about facets of the teacher that are categorized as *ambivalent or as unchanged*. Participants who held a negative perception of their teacher at the first measurement point perceived the teacher as unfair, authoritarian, or intimidating (10) or described questionable educational methods on the part of the teacher (6). After one school term involving animal-assisted education, this teacher is seen as happier, more cheerful, friendlier, and less strict. Moreover, the teacher is perceived as fairer and less disciplinarian. The teacher is noted for a heightened differentiation in teaching methods and increased sensitivity to ambient noise levels, culminating in a timelier indication of heightened noise levels to the students. Some children theorize that the canine companion serves as a sentinel, alerting the instructor to classroom issues. The presence of the dog encourages interaction on a personal level.

“R: Firstly, I used to have such earache, or something (…) had kind of scared me constantly when Mr. FBO sort of started shouting like that. But somehow, since the dog has been there, that’s no longer the case”

(F8_22, Group 2, Segment 50).

“R: Well, I noticed that Mr. FBO has become a bit more cheerful. I’ve never seen Mr. FBO laugh since [dog’s name] was there. Never. I: Before you mean? R: I never saw Mr. FBO laughing before. I: OK. R: Never. I: Yes. R: He’s been laughing since [dog’s name] came. He laughs (…) whenever he wants. I: OK. R: So, the lessons with Mr. FBO have (…) so are more (…) fun”

(F8_1, Group 2, Segment 107–115).

### 3.2. Results in Part 2: Potential of Animal-Assisted Education

The participants mention a great attraction to dogs and describe the affection they feel towards them (10). Additionally, dogs are regarded as givers and receivers of trust, love, and comfort (8), they are seen as interaction partners (2), and the human–animal relationship is characterized by its healing and empowering qualities (4). Moreover, contact with a dog is described as a privilege and unique feature of the class (1) and another child describes in detail the fascination that dogs exude.

“So, it [a dog] is a special animal, a living being in the truest sense. And that’s why I think you should also be good to animals […]. So, I think it’s special, something so special”

(F5_18, Group 2, Segment 50).

Four participants describe single situations in which the dog feels uncomfortable and for example started barking (4). Animal welfare is a central theme in animal-assisted education and these statements emphasize its relevance since animal welfare seems not to be taken care of in these situations.

Four participants identify human actions, behaviors, or attitudes that might prevent human–animal relationships or do not correspond to reality, such as when animal-assisted education is seen as a solution by the teacher but not by the students.

“Yes, he [teacher] said that the dog would be quite beneficial for me, but (…). I: You don’t see it that way necessarily. R: No”

(F8_6, Group 2, Segment 38–40).

The following section will examine the participants’ descriptions of fellow students interacting with the animal or behaving differently than usual when animal-assisted teaching is taking place (13). A striking feature of these observations of others is how detailed and astonished the participants are when describing fellow students’ interaction with the dog or their behavior in the animal-assisted setting. These observations are not consistent with previous assumptions or perceptions of the person observed, leading the participants to revise or modify their subjective theories about individual classmates. In addition, some participants (11) describe an increased intensity of feelings of fellow pupils towards the dog and other fellow pupils.

“R: As far as I’ve seen, whenever a dog has come to one of our boys, they’ve been quite careful. I: OK. R: So, they weren’t as, you know, rough as they were to other students, for instance, but rather more careful with the animal. I: Oh, really? R: Yeah, so give the dog a stroke, or whatever, but they didn’t, for example, they didn’t talk so loudly either. They didn’t yell or (…), just very quietly. I: Would you have expected this before? R: Well, with some of them, I would have, but I was still a little bit unsure with others as to whether they would show a little bit of change with the animals. I: OK. Would you say, that they were somehow different? R: Yes”

(F8_8w, Group 2, Segment 54–62).

“So, for example, F29, he is actually, actually also totally loud and such, but he is now fully concentrated, and he also asks me for help when he doesn’t understand something, and this is something that has surprised me a bit I: And this is quite new? OK R: Yes, so it was like this in fifth grade. But then we had another teacher, who always played music for us. But then, a long time passed where F29 didn’t do anything at all. And now, he makes the effort again, and that has surprised me so positively. I: When did he start doing things again? R: I don’t know, now for a month, or two. I: OK, yeah good. Can you figure out why that is, or is it something you just noticed? R: I just noticed it”

(F8_3, Group 2, Segment 48–54).

A lack of interest in the dog, a reluctance or low affinity for dogs potentially could lead to irritation—and possibly to exclusion. This is not something that can be empirically deduced from the interviews, but the following statement leads to this assumption:

“I thought (…) yes, I thought, for example, F25 (…) I thought he was giving the dog more attention. But he’s not giving him any attention at all. So, he doesn’t pay attention to the dog. So as if he wasn’t there. I: OK. R: Yes. I: You wouldn’t have thought so? R: Yeah, I just thought (…) looking more at him or something, but he doesn’t at all. He is fully focused on the work”

(F8_7, Group 2, Segment 143–147).

It is also evident that some children use interaction or looking at the dog as a way to get out of unpleasent social situations or challenging learning situations: Animal-assisted education is therefore seen as a kind of coping strategy among fellow students (2).

“R: Well, I also think that they are looking for a bit of a connection with the dog because it distracts them a bit from the work they are doing. And that makes it easier to work afterwards. I: Why do you think you can handle it better afterwards? R: Because maybe your head was somewhere else for a while and you had the chance to clear your head a bit. Thanks to the dog that was there”

(F8_18, Group 2, Segment 14–16).

Another implicit indicator of the potential of animal-assisted education is the multiple interpretative patterns with which the participants describe animal-assisted education [29]. From the participants’ point of view, there are 11 different interpretations of the interactions of dogs, students, and teachers. The pattern of interpretation, “The Teacher involves a dog for the benefit of the class” (T-D-C, 6 participants), for example, is conducive, since the teacher is seen as a caring person who is responsive to the students. The teacher works with the animal because he or she values and trusts the pupils.

“R: The dog is indeed present, and Mr. FBO gives us his deepest trust because of the dog’s presence. And the dog is, in fact, just like a child for Mr. FBO. Because, of course, he raised her, [dog’s name]”

(F8_1, Group 2, Segment 75).

“R: Yes. We have to give [dog’s name] the greatest sense of security. Out of everyone in the class, she is our (…), she’s our guest. And after all, Mr. FBO trusts in us to take care of her”

(F8_1, Group 2, Segment 77).

The perception that the students can influence the teacher’s behavior through the dog (C-D-T) or that animal-assisted education primarily benefits the teacher (D-T, 1 participant) is less conducive or neutral.

“I think it’s also quite nice for her if she doesn’t have to leave [dog’s name] at home, she still can watch over her as well while doing lessons”

(S5_20, Group 2, Segment 42).

This shows the relevance of communicating the teacher’s intention, and thus the opportunity to show the teacher’s appreciation of the students and the dog.

## 4. Discussion

The discussion’s initial segment will focus on the discussion of research findings regarding the current state of the field. In the second part of the discussion, the results will be examined concerning role theory, thus creating an explanatory approach to the perceived effects of animal-assisted education in the school setting.

### 4.1. Discussion concerning the State of Research

The results of the qualitative exploratory survey about the influence of animal-assisted education on social participation and social climate both confirm and complement findings about animal-assisted interventions. Animal-assisted education is associated with reducing maladaptive strategies, diminishing aggressive behavior, enhancing the child’s emotion regulation, and fostering a tranquil and relaxed atmosphere within the school environment through animal interaction [12,13,14,15,16,17]. Our findings as presented above confirm that results of previous research can also be found in the pupils’ perception of animal-assisted intervention. The participants perceived a positive influence of animal-assisted interventions on pro-social behavior, mood, and caring behavior in students, while they described school dogs as individuals to whom students form attachment-like relationships. The latter reflects the findings of Julius et al. [23], who developed the attachment theory based on neurobiological and psychological studies of human–animal relationships. The role theory is an approach to describe and justify social behavior while considering the social setting [36]. Therefore, these results are drawn upon in the second part of the discussion and contextualized concerning role theory.

Additionally, we observe that school dogs exert a discernible influence on the social dynamics within a school class. The students and teacher share the goal of caring for the dog and taking responsibility for its welfare. The school dog thereby constitutes a shared interest, whereby exclusionary group structures can be reduced and class cohesion and class identity can be reinforced. This strengthens the bond between pupils and between the teacher and pupils, and thus animal-assisted education becomes an opportunity for social participation. This finding is consistent with the findings that social participation is promoted by common characteristics of pupils [7]. In animal-assisted pedagogy, the common and thus unifying feature is the fact that one is taught with a dog, that one is interested in the dog, takes responsibility for it, and cares for it. At the same time, it can be suspected that social exclusion could occur if children do not show interest in the animal over a longer period. The phenomenon of shared goals and shared interest concerning a school dog is to be examined in the second part of the discussion concerning role theory.

In our investigation, we reveal that the mutual perception of the pupils in a school class as well as the perception of the teacher is influenced by animal-assisted pedagogy. The way fellow students and the teacher interact with the dog is observed by other children. This allows children and teachers to transform previous individual unfavorable attributions. Our findings also suggest that the presence of a dog could potentially facilitate alterations in stereotypical role structures and mitigate the labeling effect associated with children having special educational needs. This effect, as proposed by Henke et al. [9], serves as a predictor for exclusion. Our data indicate that fellow students are perceived as friendlier, more approachable, and more considerate. Teachers are seen as fairer, calmer, more competent, and easier to trust. These results regarding the altered mutual perception will also be examined in the second part of the discussion concerning role theory, since social roles are closely linked to social expectations and, consequently, mutual perception. The study did not investigate whether the presence of the school dog changed the teacher’s perception of the students. The teacher’s behavior towards a student significantly influences the behavior of other children toward that student [47]. If the teacher perceives a student to be (unexpectedly) caring towards the dog, this could affect the teacher’s attitude towards the student and, in turn, influence other students’ interactions with that student.

Our findings reveal numerous positive impacts on inclusive structures through involving a dog in teaching but also show some negative tendencies, as being taught with a school dog is sometimes stressful for students. This occurs when pupils are concerned about the welfare of others; for example, if they have previously expressed concerns or fears. Situations where animal welfare is not ensured also become distressing experiences for the children. The welfare of animals represents a fundamental and yet under-appreciated pillar of animal-assisted education, which we will revisit in our conclusion. 

Moreover, as our data indicated, a mismatch in the intensity and quality of direct interaction with the dog can cause jealousy. Negative consequences of animal-assisted education also become obvious when pupils are entrusted with too much responsibility for the animal. At this point, the teacher’s responsibility for a successful animal-assisted pedagogy becomes visible.

We show that the participants justified the impact of a school dog based on two different explanations. The first explanation our research hints at is a thesis that is currently common in the research field: Behavior and well-being improve because of the dog’s presence. The second explanation is new, indicating that the children consciously choose to take responsibility and care for the animal. The presence of a school dog seems to change social roles on the one hand, and on the other hand, new social roles emerge, such as the “role of caregiver for the dog”; in short, the social role of “the, caregiver”. Below, we shall elaborate on how animal-assisted education influences social roles and how a new social role emerges, based on the methodological basis of symbolic interactionism [34,35] and with reference to the role theory of Krappmann [36], which was briefly introduced in Section 2.1.

### 4.2. Discussion concerning Role Theory and Explanatory Approach to Animal-Assisted Education in School

The results of this study indicate that students whose teaching involves a dog take on another social role, the role of the *caregiver* (for the dog). In this role, humans’ emotional needs for attachment and care are met, leading to a change of norms in the classroom setting. Whereas previously, individual objectives had to be achieved, now there is a shared desire to take care of the dog (participants are observed collectively preparing a bed and water or reminding each other to be more considerate of the dog). This facilitates the negotiation processes between individuals because they share the same objective when they encounter each other in the role of the caregiver (*role action*), a role which is voluntarily chosen (*role-taking*) and implemented similarly, since the participants agree about the dog’s needs (*role-making*). 

The regular presence of a school dog also influences the identity of a school class, since social roles shape identities [36]. Instead of exclusionary group structures, the common identity as a class that cares for a school dog is shared. Animal-assisted education depends on those involved being mindful of the dog’s needs. As mentioned earlier, students perceive themselves and others differently when interacting with the dog. In doing so, previous individual and stereotypical perceptions of a person are changed, which can be explained with the use of the role theory. Two typical changes in perceptions are as follows. First, a child that previously struggled to regulate their own emotions now interacts lovingly with the dog and behaves more considerately in the role of caregiver. Here, the individual attributions are altered through observing the child in the role of the caregiver. Second, the boys who previously appeared aloof to conform to the stereotypical image of masculinity now communicate with the dog in an affectionate manner, which leads to individual and stereotypical perceptions being altered. Interpersonal interactions are also influenced by changing perceptions of others, since even if a person does not engage in the role of carer, this role belongs to their repertoire of roles, influencing the self-image and expectations of each other [36]. In terms of self-image, the pupils have more confidence in their ability to act considerately and regulate their emotions. Regarding the expectations of others, if a child interacts considerately and kindly with the dog, there will be an increased expectation that they will interact nicely with other children as well. Krappmann’s role theory [36] covers the areas of *role-taking*, role assumption (*role-making*), and acting in social roles (*role action*), as well as the area of *norms* that influence role action. Norms are influenced in two ways. On the one hand, caretaking, mindfulness, and consideration are established as new norms in the animal-assisted setting, as a constitutive framework of animal-assisted pedagogy. On the other hand, the norms of the school setting, such as the hierarchical difference between teacher and pupil, selection, and performance, do not apply to the dog, whereby the dog as an actor partially relegates existing norms to the background.

It can be assumed that these changes in social roles are transferable to other group settings, such as group settings in social work or therapeutic settings. In the school setting, it can be considered that the attitude of the teacher is crucial to the success of inclusion and animal-assisted education. Teachers’ interactions with students influence how other children interact with these students and whether they experience social participation [47]. In the same way, the pupils’ interaction with the dog could depend on the teacher’s behavior; the theory of learning from a model [48] suggests this. Therefore, teachers can and should combine both child development through animal-assisted pedagogy and animal welfare in inclusive settings. An inclusive attitude on the part of the teacher towards the dog and the person is fundamental to this. If the needs of the dog are recognized, valued, and safeguarded, and animal-assisted education is developed on this basis, then this can serve as an example of respectful and considerate interpersonal relationships. 

## 5. Limitations

The research project [29] only covered four school classes, so the results cannot be generalized. Additionally, the theoretical concept requires further development and review, but should represent an initial explanatory approach from the perspective of educational science for the school setting. A distinct weakness of the research has been that it lacked a multi-perspective approach; the perspectives of the teacher, not to mention the dog, were missing. For instance, the dog’s perspective should be explored by involving dog trainers to safeguard aspects of animal welfare and to compare whether the students’ statements about caretaking to meet the dog’s needs are reflected in reality. Research always represents only a small segment of reality, yet there is a special obligation for researchers in the field of animal-assisted interventions to ascertain the setting and burdens and implications of all participants as comprehensively as possible. 

## 6. Conclusions

Our research aims to assess the perceived effects of animal-assisted education on students’ social participation and the overall social atmosphere within school classrooms. The results of the present study show that the involvement of a dog can influence group structure and social participation.

- The presence of a school dog appears to influence social roles. On one hand, existing social roles are altered, while on the other hand, new social roles emerge, particularly the “Caregiver” role, in which students feel the need to take responsibility for and care for the animal. As a result, mutual perceptions between students and between students and their teachers improve, leading to a more positive image of each other.
- Animal-assisted pedagogy leads to a reduction in stereotypes and individual prejudices and a transformation of norms within the school environment. Whereas previous social norms led to exclusion or differentiation from others, there is now a shift towards more appreciative and respectful interactions.
- Animal-assisted pedagogy presents an opportunity for social participation through shared interests and goals among students and between students and their teacher. Previously, differences were employed as markers of identity, but now, the shared experience of being taught with the assistance of an animal becomes the common bond for identification. However, the potential risk of exclusion stemming from disinterest needs to be further explored.
- Animal-assisted pedagogy fosters improved pro-social behavior, mood, and student empathy, highlighting the strong attachment-like relationships cultivated with school dogs, while jealousy and overwhelm may pose a threat to the positive effects.
- Animal welfare is relevant to both the well-being of animals and to educational processes. Reinforcing and promoting care for the animal (caretaker’s role) establishes the foundation for the effects of animal-assisted interventions, while simultaneously practicing animal welfare.

The latter is to be understood as a central conclusion for research on human–animal relationships, as well as for the practice and research of animal-assisted education. The examination through the lens of role theory underscores the profound significance of the “caregiver” role in the context of animal-assisted pedagogy. It is evident that the welfare of animals not only holds intrinsic value for their well-being but is also constitutive for the promotion of social participation through animal-assisted interventions. 

This leads to the conclusion that animal-assisted pedagogy should be based on the pillars of inclusive pedagogy: when a teacher regards the dog as an equal partner, allowing the dog to autonomously determine its preferred level of interaction, including the option to retreat to a secluded and secure resting area (in line with the recommendations of the Quality Network School Companion Dogs e.V. [40]), this practice becomes a powerful means of conveying the dog’s needs and boundaries. This leads to the respectful and caring treatment of the dog, serving as a role model for how to interact with oneself and with others. The current goal in the development of animal-assisted pedagogy should be to see the dog as a shaping actor, a subject that shapes the situation by its mere presence. Animal-assisted interventions based on this contain both: animal welfare and conditions for successful animal-assisted pedagogy to promote social participation and social climate.

## Data Availability

The data presented in this study are available on request from the corresponding author. The data are not publicly available because of the privacy policy used, which was signed by the participants.
